# An arcane cardiomyopathy

**DOI:** 10.1007/s12471-014-0603-3

**Published:** 2014-09-26

**Authors:** J. R. T. C. Roelandt

**Affiliations:** Thoraxcenter, Erasmus Medical Center, s’Gravendijkwal 230, 3015 GE Rotterdam, the Netherlands

Last February, the Lancet and the New England Journal of Medicine almost simultaneously published two interesting case reports on an arcane cardiomyopathy [1, 2]. The New York Times drew attention to these papers in their Health Section as ‘A Medical Mystery Solved’.

A 55-year-old man is described. He was hospitalised for severe heart failure after he had gone from doctor to doctor seeking a diagnosis for his many puzzling ailments. His left ventricular ejection fraction was 25 % and his coronary arteriogram normal. In 2010 his ceramic artificial hip was replaced by a metal-on-metal type, which to the doctors had seemed unrelated to his many symptoms (heart failure, deafness, almost blind, hypothyroidism, and fever). However, a doctor of the team in Marburg (Germany) remembered an episode of Dr. House that he watched on the Fox TV show (also broadcast in Germany and in the Netherlands), on a mysterious illness caused by metal poisoning from an artificial metal hip. When they measured the patient’s blood serum cobalt level it was 1000 times higher than normal. Radiography showed metal debris near his metal hip. After chelation therapy and replacement of his metal hip with a ceramic type, his condition improved and his ejection fraction rose from 25 to 40 %. Cobalt intoxication was the most likely cause of the heart failure and the many other ailments of this patient [1].

The other report describes a 59-year-old woman who underwent heart transplantation for severe heart failure, also after a few years of puzzling complaints and symptoms. In her work-up all rare causes of her medical history were considered but none showed up. She had undergone right total hip replacement 4 years previously and left total hip replacement 3 years previously, both with a metal prosthesis. Sometime after her heart transplantation her orthopaedist unexpectedly found her blood serum cobalt level to be 300 times higher than normal on a routine check-up. A year later both artificial hips were replaced with prostheses with a polyethylene liner. Her cobalt levels decreased and her condition improved. The most likely cause of her symptoms and unexplained heart failure was cobalt intoxication [2].

Since 2006, there is an increasing number of publications on patients with problems after a metal-on-metal hip arthroplasty. As a result of the wear over time at the bearing surfaces, chromium and cobalt are commonly found in the blood and urine of these patients. In the vicinity of the prosthesis, the metal ions can cause metallosis resulting in giant cell infiltration, fibrosis and solid pseudotumours [3–6]

In 2010, Tower was the first to draw attention to the neurological and cardiac adverse effects of chronic systemic cobaltism [7]. Subsequently, a few case reports have been published on cobalt cardiomyopathy, one with fatal outcome [1, 2, 8–10].

When I was starting my fellowship in cardiology at the University of Leuven (B) in 1966, my mentor, Professor H. Kesteloot published a series of patients with severe heart failure. All were heavy beer drinkers. Strangely enough, these patients had a large pericardial effusion and polycythaemia, two findings which had not been reported before in alcoholic cardiomyopathy. Also, after tapping the fluid, their heart size was not significantly enlarged. The syndrome was presented as a new form of cardiomyopathy in chronic beer drinkers [11].

Early in 1967, there was a request from the FDA as to whether they could come to Leuven to examine the files of these patients. After studying the data, they told us the story of the Quebec beer drinkers, about the similarities between the two groups and that cobalt intoxication was the most likely cause [12, 13].

Between August 1965 and April 1966, 48 patients, all heavy drinkers, were admitted to the Department of Cardiology of the Laval University of Quebec (Canada) and 20 of them had died. All patients were drinking beer of the Dow Brewery. This brewery had been adding cobaltous sulphate to their beer since July 1965 to stabilise the foam layer. Analysis showed that that brand of beer contained 10 times more of the chemical than other beers. The rapid decrease of the foam layer was an increasing problem due to the washing of the glasses with detergents (women increasingly frequented bars in the 1960s and lipstick could not be removed from the glasses by just rinsing in plain water). In the same period, the chemical had also been added to the beer in breweries in Omaha (Nebraska, US). In three hospitals, 64 patients were admitted of whom 40 died [14].

Together with my co-fellow J. Willems we traced the ‘Leuven’ patients, convinced them to come to the clinic for clinical re-examination and blood analysis, ECG and asked them the brands of beer they were drinking to test for their cobalt content. All patients drank beer containing cobalt [15]. The similarities of the medical history of the patients in Quebec, Omaha and Leuven led to the conclusion that the aetiological role of cobalt could be accepted as proven. The synergistic toxic effect of alcohol and cobaltaemia was later confirmed in experimental studies [13, 16].

The addition of cobaltous sulphate (or cobaltous chloride) to beer to stabilise the foam layer was patented and distributed by A. Jorgenson Laboratories in Copenhagen. It was removed from the market in 1967.

In the recent edition of his textbook Heart Disease, Braunwald mentions that no single case of the acute and severe form of cobalt cardiomyopathy has been reported since [17].

Some questions remain. Why did none of our patients die and why did only a small fraction of the population drinking cobalt containing beer develop heart disease? This could be due to an abnormal sensitivity and/or to a synergic action of different factors in certain individuals. Nutritional deficiencies definitely played a crucial role. All patients were anorexic, had a lower economic status and had an inadequate protein intake. However, our patients regularly consumed hard boiled eggs (barkeepers in Belgium often offered boiled eggs to their loyal customers) containing a lot of sulfhydryl amino acids (cysteine, methionine), which form an irreversible chelate with cobalt and effectively eliminates cobalt.

The serum blood levels of cobalt in arthroplasty patients and in cases of industrial exposure to cobalt are much higher than they were in the beer drinkers. Why, then, is the incidence of a toxic cardiomyopathy so low? In general, the nutritional status of these patients is good and the alcohol intake in the large majority limited. Cobalt blocks the oxidative metabolism at the mitochondrial level resulting in cellular dysfunction or death, but the factors which determine cardiac toxicity in chronic cobaltaemia are certainly multifactorial and largely unknown.

Many patients with a metal hip prosthesis are in the older age group in which heart disease is more prevalent. Cardiologists should be aware of the potential toxic and systemic adverse effects of cobalt (tinnitus, vertigo, deafness, visual changes, hypothyroidism, neuropathy and heart failure) as chronic elevated cobaltaemia may have an additional effect on a co-existing myopathy.

Over 10,000 patients have received a metal-on-metal hip prosthesis in the Netherlands since 1999 and about 10–15 % have been replaced (Bom LPA. Personal communication 2014). In April 2010, the UK Medical Products and Healthcare Devices Regulatory Agency published the first medical device alert (and an update in 2012) and recommended to monitor all patients with a metal-on-metal hip arthroplasty for cobalt toxicity symptoms [18]. In 2008 the Dutch Orthopaedic Association started a national registry of all hip implants, the LROI (Landelijke Registratie Orthopedische Implantaten). Because of the apparent side effects, the Dutch Orthopaedic Association strongly advised their members against the use of metal-on-metal hip prostheses and advised yearly follow-up of all patients with such a prosthesis. Patients with a serum blood cobalt level > 40 nmol/L require close surveillance for signs and symptoms of cobalt toxicity [19].
